# Indications for the Induction of Premature Labor

**Published:** 1887-09

**Authors:** 


### *INDICATIONS FOR THE INDUCTION OF PREMATURE LABOR.*

A few weeks ago, Dr. George T. Harrison read an admirable paper before the New York County Medical Association on the *Indications for the Induction of Premature Labor*, which is worthy of careful study. In general terms it may be said that the object sought in this operation is to give a better prognosis in those cases in which the further continuance of pregnancy, or childbirth at term, involves great danger to mother or child, or both, by artificial termination of pregnancy at a time when the fœtus is capable of living outside the uterus. As the chief danger to the mother, in the performance of the operation, is not, as formerly supposed, the mechanical irritation of the uterus, but septic infection, it may be affirmed that, if proper antiseptic precautions be observed, this danger is an avoidable one.

The first and most important indication for the operation is furnished by pelvic deformity of a moderate degree. Theoretically this indication is clear enough, but in practice several difficulties may arise in its fulfillment: *First*, our means for pelvic measurements are imperfect, and only enable us to approximate to correctness. *Second*, the exact period of gestation may be very difficult to determine, in certain cases deviating somewhat from the normal. Irregularity especially occurs in cases of contracted pelvis, from the fact that the head is prevented from entering the pelvic cavity, and thus certain signs of pregnancy are modified. But, having fixed the date of gestation as accurately as possible, we must next decide in what week of pregnancy the induction of artificial labor is indicated. The earlier the labor is induced, so much the less danger of injuring the soft parts of the mother, and so much greater the chances that the child will be born alive; but, at the same time, the prospect of the child's continuing to live diminishes in proportion to its prematurity. On the contrary, the later the labor is induced, the greater the danger to the mother and child, but the better the prognosis for the survival of the child if living. The problem, then, is to choose such time when the child could be born without injury to itself or the mother. To do this we must get an accurate idea of the sizes of the pelvis and the child's head.

In the flat pelvis, the deformity most frequently met with, we have simply to ascertain the measurement of the conjugate diameter. In the equally contracted pelvis the problem is much more intricate. Here we must introduce the whole hand into the vagina, in order to form an idea of the pelvic cavity, and errors are difficult to avoid. To determine the size of the child's head also presents many difficulties. Schrœder showed by careful measurements that the head is larger from the twenty-eighth to the fortieth week of gestation than was generally supposed to be the case. But it must be remembered that the head of immature children are much more compressible than those of children at term. Other criteria which may serve as guides are the facts that large and powerful women, as a rule, give birth to large children, and that the weight of the child increases with the age of the mother and with the number of preceding births. The transverse diameter of the heads of the children of young primiparous women is relatively small; while the contrary is true in multiparæ who are comparatively old. The method proposed by Frankenhäuser and Roth, of determining the relation between the head of the fœtus and the pelvis of the mother, is worthy of trial. This consists in passing the child's head into the pelvis, and, by means of the fingers in the vagina, ascertaining if it be possible for the head to descend below the brim. This examination should be repeated every eight days, and operative interference should be resorted to only when the descent of the head below the brim seems no longer possible.

As to the degree of pelvic contraction which justifies a resort to the induction of premature labor, Dr. Harrison believes that in the simple flat pelvis a conjugate diameter of 7.5 cm., or 7 cm. in very exceptional cases, should be the extreme limit. In the equally contracted pelvis, when the shortest diameter is at least 8 cm., it may be said that the operation is justifiable. As a rule, the thirty-sixth week of pregnancy is to be selected, and only exceptionally should the term of gestation be anticipated by operating in the thirty-fourth week.

An indication for inducing premature labor is also given by certain severe diseases which endanger the mother's life and are amenable to no other treatment; while, on the other hand, either a cessation or an amelioration of the symptoms may be expected with the termination of pregnancy. Among such affections is uncontrollable vomiting appearing late in pregnancy. Occasionally it is so severe that operative interference is imperatively demanded. Another disease which may indicate the operation is nephritis; though a distinction must be made between cases in which the anatomical and functional renal disturbances are evoked by the pregnancy, and those in which the disease is an interstitial or parenchymatous nephritis, which either existed prior to conception or was produced during the course of gestation. As to the induction of premature labor to prevent eclampsia, Dr. Harrison believes the indication clear and decided when the urine shows a constantly increasing quantity of albumin and fatty degenerated cells, and when all other resources prove unavailing. Again, excessive œdema and transudation into the serous cavities may demand the operation. To diagnosticate between the two forms of nephritis referred to, *nephritis gravidarum* and *nephritis in gravitate*, is no easy task. The clinical phenomena are so nearly alike that the previous history of the case and the course it took can alone decide as to which form is present in a given case. It has been abundantly shown that in chronic nephritis in pregnant women, there is a great tendency to abortion and premature labor, and not so much to attacks of eclampsia.

A third indication for the induction of premature labor in the interest of the child, is met with in certain cases in which experience has shown that the children died at a certain date of gestation, when this time was not far removed from the end of pregnancy. Some cases of *nephritis gravidarum* come under this category. If syphilis be the cause of death of the fœtus, the artificial interruption of pregnancy Dr. Harrison thinks is contra-indicated; for, according to Kassowitz, in latent syphilis of the father or mother, the death of the fœtus takes place in each ensuing pregnancy somewhat later; so that in successive pregnancies these are first abortive fruits, then immature macerated, then permaturely born macerated, and finally mature, but diseased children; still later, mature healthy children. On the other hand, we gain nothing by the artificial induction of pregnancy before the expected time of the death of the fœtus, for the child is already diseased, and may be considered as lost. In cases, however, in which chlorosis or anæmia of the mother, or changes in the umbilical cord or placenta, have caused the death of the fœtus, the induction of premature labor before the critical period may be the means of securing a living child.

It is rare that diseases of the heart or lungs give an indication for this operation, though they occasionally do. Lastly, an indication for the induction of premature labor is furnished by those dangerous and incurable diseases of the mother which would probably cause death before the end of pregnancy, in order to avoid the Cæsarean section *post mortem* or *in articulo mortis*.—*Journal of the American Med. Association.*

